# Strength Lies in Diversity: How Community Diversity Limits *Salmonella* Abundance in the Chicken Intestine

**DOI:** 10.3389/fmicb.2021.694215

**Published:** 2021-06-15

**Authors:** Adriana A. Pedroso, Margie D. Lee, John J. Maurer

**Affiliations:** ^1^Department of Population Health, University of Georgia, Athens, GA, United States; ^2^Department of Biomedical Sciences and Pathobiology, Virginia Polytechnic Institute and State University, Blacksburg, VA, United States; ^3^Department of Animal and Poultry Sciences, Virginia Polytechnic Institute and State University, Blacksburg, VA, United States

**Keywords:** *Salmonella*, chicken – broiler, intestine, diversity, *Clostridia*

## Abstract

The transfer of the intestinal microbiota from adult to juvenile animals reduces *Salmonella* prevalence and abundance. The mechanism behind this exclusion is unknown, however, certain member species may exclude or promote pathogen colonization and *Salmonella* abundance in chickens correlates with intestinal community composition. In this study, newly hatched chicks were colonized with *Salmonella* Typhimurium and 16S rRNA libraries were generated from the cecal bacterial community at 21, 28, 35, and 42 days of age. *Salmonella* was quantified by real-time PCR. Operational taxonomic units (OTUs) were assigned, and taxonomic assignments were made, using the Ribosomal Database Project. Bacterial diversity was inversely proportional to the *Salmonella* abundance in the chicken cecum (*p* < 0.01). In addition, cecal communities with no detectable *Salmonella* (exclusive community) displayed an increase in the abundance of OTUs related to specific clostridial families (*Ruminococcaceae*, *Eubacteriaceae*, and *Oscillospiraceae*), genera (*Faecalibacterium* and *Turicibacter*) and member species (*Ethanoligenens harbinense*, *Oscillibacter ruminantium*, and *Faecalibacterium prausnitzii*). For cecal communities with high *Salmonella* abundance (permissive community), there was a positive correlation with the presence of unclassified *Lachnospiraceae*, clostridial genera *Blautia* and clostridial species *Roseburia hominis*, *Eubacterium biforme*, and *Robinsoniella peoriensis*. These findings strongly support the link between the intestinal bacterial species diversity and the presence of specific member species with *Salmonella* abundance in the chicken ceca. Exclusive bacterial species could prove effective as direct-fed microbials for reducing *Salmonella* in poultry while permissive species could be used to predict which birds will be super-shedders.

## Introduction

*Salmonella* is a γ-proteobacter capable of colonizing the gastrointestinal tract of many animal species (Sanchez et al., 2002). The evolution of the genus *Salmonella* involved the acquisition of a pathogenicity island encoding a type III secretion system that mediates cell invasion (Baumler et al., 1998). This pathogenicity island (SPI1) is central to *Salmonella*’s ability to cause disease in many animal species (Galan and Curtiss, 1989; Watson et al., 1998; Lichtensteiger and Vimr, 2003) but is primarily responsible for inducing the inflammation associated with gastroenteritis (Jung et al., 1995; Hapfelmeier et al., 2004). By eliciting this inflammation, *Salmonella* infection creates a metabolically favorable environment for the pathogen that results in improved growth in the intestine (Winter et al., 2010; Thiennimitr et al., 2011; Rivera-Chavez et al., 2016).

*Salmonella* infection causes the most severe symptoms in young mammals (Wray and Wray, 2000), and chickens exhibit symptoms with *Salmonella* if chicks are infected *in ovo* or shortly after hatch (Wray and Wray, 2000). If challenged at 2 days of age, many chicks fail to exhibit any symptoms even though nearly all of the birds become colonized and shed the organism for 4–6 weeks (Cheng et al., 2015). The microbiota of the hatchling evolves quickly so that by 3 days posthatch, several dozen distinct bacterial species inhabit the chicken gastrointestinal tract, with γ-proteobacter accounting for ∼2% of the total species population (Lu et al., 2003a; Pedroso et al., 2016). Within the next 3 weeks, species diversity in the intestine increases with a succession of bacterial species in the ileum and cecum and γ-proteobacter become a minor component of the intestinal community (Lu et al., 2003a). The community composition of the chicken ileum and cecum are similar within the first week post hatch comprised primarily of *Firmicutes* related to the *Lactobacillales* and *Clostridiales* (Gong et al., 2002; Lan et al., 2002; Lu et al., 2003a; Zhu and Joerger, 2003). The ileal and cecal communities become segregated quickly, the composition of each becomes unique, and community diversity peaks in broiler chickens near the time when they are processed at 49 days of age (Lu et al., 2003a). Chicks exposed to a mature intestinal microbiota at hatch rapidly develop high community diversity (Lee et al., 2006; Pedroso et al., 2016) and are resistant to *Salmonella* colonization (Nurmi and Rantala, 1973; Nurmi et al., 1992; Nakamura et al., 2002). In fact, the microbiota from chickens, as young as 21 days of age, seeded in newly hatched chicks dramatically reduces *Salmonella* abundance in 1-week old layer chickens (Varmuzova et al., 2016). These findings are the basis of the concept and practice of competitive exclusion.

Humans, regardless of age, can present gastroenteritis upon consumption of *Salmonella*-contaminated water, milk, or food. While susceptible mouse strains are commonly used as an animal model for understanding *Salmonella* pathogenesis, these infected animals present a lymphoid-associated enteric fever instead of gastroenteritis (Santos et al., 2001). However, mice administered streptomycin, prior to challenge, develop inflammation of the colon with *Salmonella* infection (Hapfelmeier et al., 2004). Streptomycin treatment decreases abundance of *Firmicutes* and increases *Salmonella* abundance in challenged mice (Sekirov et al., 2008) indicating that the composition of the intestinal microbiota therefore has a profound effect on pathogen behavior. A decline in intestinal species diversity favors enteropathogen colonization (Antharam et al., 2013; Lone et al., 2013; Stanley et al., 2014; Zhang et al., 2015) and disease (Antharam et al., 2013; McMurtry et al., 2015; Rodriguez et al., 2015; Singh et al., 2015). Why are chicks protected from enteropathogen colonization when seeded at hatch with intestinal microbiota from adults versus juveniles? What changes in the intestinal microbiota as animals age causes this colonization resistance? Microbiome diversity may ensure the presence of sufficient competitors or antagonists to block pathogen colonization and persistence.

In order to study these hypotheses, a molecular ecology approach was used to reveal the intestinal community structure relative to *Salmonella* abundance in chickens. Community diversity and abundance of some species correlated with *Salmonella* abundance in the chicken cecum. Several *Firmicutes*, particular clostridial species, were positively associated with *Salmonella* abundance, but others correlated with low *Salmonella* abundance. It appears that species diversity may be key to understanding pathogen exclusion in the intestine.

## Materials and Methods

### *Salmonella* Colonization

A total of 100, 1-day-old, specific pathogen-free, white leghorn chickens (Charles River Laboratories; Wilmington, MA, United States) were placed in one of five HEPA-filtered, isolator units (20 birds per unit). Each unit has wire mesh floors to reduce re-exposure due to coprophagy, and received feed and water *ad libitum* up to 42 days of age. Chick box liners and the inside of isolator units were swabbed for *Salmonella* with milk-soaked, 3M^TM^ Sponge Stick (3M; St. Paul, MN, United States) as previously described (Liljebjelke et al., 2005). Birds and their environment were culture-negative for *Salmonella* on the day of placement of chickens in isolator unit. Chickens were reared on a commercial, non-medicated, pelleted, starter feed throughout the course of the study. At 2 days of age, chicks were orally inoculated with 1.1 × 10^6^ CFU/0.1 ml of *Salmonella* Typhimurium SL1344. *Salmonella* inoculum was prepared by streaking SL1344 onto Tryptic Soy Agar (Thermo Fisher Scientific; Pittsburg, PA, United States) which was subsequently used to inoculate 5 ml Luria-Bertani (LB) broth (Provence and Curtiss, 1994) in sterile, capped, 13 × 100 mm glass tubes (Thermo Fisher Scientific). The broth culture was incubated overnight, static at 37°C. The overnight culture was diluted 1/10 in sterile saline. The *Salmonella* challenge inoculum was serially diluted 10-fold in buffered saline gelatin (Provence and Curtiss, 1994) and plated onto LB agar to determine bacterial cell density. The number of birds per unit was maintained at a stocking density reflective of commercial standards; culling birds periodically as they grew to maintain this stocking density. At 21, 28, 35, and 42 days of age, one bird from each of the five isolator units was collected (*n* = 5), euthanized and the ceca were aseptically removed. The number given to each sample (1–5) corresponds to the isolator unit from which the bird was collected. Cecal contents were collected and homogenized in pH 7.0 phosphate-buffer saline (1:10 w/v). Between days 35 and 42, one bird died unexpectantly (isolator 5) and therefore the last time point only had four subjects, instead of five, left for analysis.

### DNA Extraction

Bacterial cells, present in the cecal contents of 19 samples, were lysed using beads, solution 1 and IRS of Mo Bio Soil DNA extraction kit (Mo Bio Laboratories Inc., Carlsbad, CA, United States) by vortexing at maximum speed for 40 min (Lu et al., 2003b). Lysates were treated with sodium dodecyl sulfate (0.5%) and proteinase K (0.1 μg/ml) and incubated at 37°C for 30 min. Samples were extracted twice with an equal volume of phenol-chloroform-isoamyl alcohol (25:24:1) and once with chloroform-isoamyl alcohol (24:1). DNAse-free, RNAse (20 μl) was added to each sample and incubated at 37°C for 15 min. DNA was concentrated with a 0.6 volume of isopropanol, and the DNA pellet was resuspended in sterile water. The quality and quantity of DNA was assessed by agarose gel electrophoresis.

### qPCR

Quantitative PCR was used to determine the amount of *S*. Typhimurium in cecal contents. A 5-μl aliquot of the lysate was diluted 1:4 in TE buffer [10 mM Tris, 0.1 mM EDTA (pH 8.0)] and used as a template in the qPCR assay. qPCR was performed using SYBR green master mix (Bio-Rad, Hercules, CA, United States) and the MJ Research Chomo4 real-time 4-color 96-well PCR system (Bio-Rad). qPCR data was analyzed by the relative standard curve method (Fey et al., 2004). Serial diluted DNA from *S.* Typhimurium SL1344 was used as a standard. The virulence gene *invA* (Daum et al., 2002) was used as the target amplicon to estimate *Salmonella* abundance and the gene *ttr* (Malorny et al., 2004) was used as an internal reference for data normalization.

### PCR Amplification of Cecal 16S rRNA Libraries

The bacterial primers 27F YM + 3 and 515R-NK (5-CCG CNG CKG CTG GCA C-3), targeting the regions V3 and V6, were used. The primer 27f-YM3 is four parts 27f-YM (5-AGA GTT TGA TYM TGG CTCA G), plus one part each of primers specific for the amplification of *Bifidobacteriaceae* (27f-Bif, 5-AGGG TTC GAT TCT GGC TCA G), *Borrelia* (27f-Bor, 5-AGA GTT TGA TCC TGG CTT AG), and *Chlamydiales* (27f-Chl, 5-AGA ATT TGA TCT TGG TTC AG) sequences (Acosta-Martinez et al., 2008; Garcia et al., 2011). The primers were synthesized with a sequencing adaptor and a specific 8-nucleotide barcode (Hamady et al., 2008) and were a gift from Dr. William Whitman (University of Georgia). A 10 μl PCR mixture was prepared with 20 mM of each primer, 100 ng of DNA template and 9 μl of Platinum Taq DNA Polymerase (Invitrogen, Carlsbad, CA, United States). PCR amplification of the bacterial 16S rRNA genes was conducted after an initial denaturation at 95°C for 3 min followed by 20 cycles of denaturation at 94°C for 30 s, annealing at 60°C for 30 s, and extension at 68°C for 60 s. The final extension was carried out at 68°C for 4 min. PCR amplifications were done using Idaho Rapid Cycler thermocycler (Idaho Technology). DNA extracted from *S.* Typhimurium SL1344 was used as a control. PCR products were visualized by electrophoresis on 1% agarose gels, stained with SYBR Green Dye (Invitrogen) and ∼550 bp amplicons were excised from the gel. Amplicons obtained from 3 replicates of the same samples were pooled together. Products were purified from the agarose gel initially using the Qiagen QIAquick Gel Extraction Kit (Qiagen, Valencia, CA, United States), followed by the Agencourt AMpure magnetic beads (Beckman Coulter, Brea, CA, United States). Purified amplicons were resuspended in water, and the quality of the fragment was assessed by agarose gel electrophoresis and the concentration was measured with a Beckman DU640 spectrophotometer (Beckman Instruments, Fullerton, CA, United States). Barcoded 16S amplicon samples were submitted to the University of Georgia Genomics Facility for pyrosequencing using a 454 GS-FLX Titanium sequencing in accordance with established methods.

### Processing, Assembly, and Analysis of Cecal 16S rRNA Libraries

All sequence processing was performed using MOTHUR software version 1.37.1 (Schloss et al., 2009). Sequences containing more than eight homopolymers nucleotides, and mismatched or ambiguous bases were removed. High-quality sequences were aligned against the SILVA database. UCHIME software was used to identify and remove chimeric sequences (Edgar et al., 2011). Operational taxonomic units (OTUs) were assigned at a 97% identity using the furthest-neighbor algorithm, and taxonomic assignments were made using the Ribosomal Database Project taxonomy – RDP (Cole et al., 2009). The error rate was assessed using the control sample, and the group was eliminated from our dataset for subsequent analysis (Schloss et al., 2009). Representative sequences of each OTU were classified using BLASTN (Altschul et al., 1990). All 16S rRNA sequence data is publicly available through National Center for Biotechnology Information (NCBI) the GenBank database under accession numbers KX913959 to KX914443. Rarefaction curves were produced as described by Hughes et al. (2001). The diversity indexes Chao, Shannon, Inverse Simpson and Smith Wilson Evenness indexes were calculated using MOTHUR. Chao index estimates the number of species (OTUs) comprising the microbial community, Shannon index determines how uniformly 16S rRNA sequences are spread into the different OTUs (Hill et al., 2003), and the Simpson index is an indication of the richness in a community with a uniform evenness that would have the same level of diversity. The inverted Simpson index was used to ensure that an increase in the reciprocal index reflects an increase in diversity (Magurran, 1988). A Venn diagram representing shared and unique OTUs was drawn for cecal communities with the two highest (1.4 × 10^9^, 1.7 × 10^9^ CFU/g) and lowest (PCR-negative) *Salmonella* abundance; representing permissive and exclusive communities, respectively (Shade and Handelsman, 2012).

### Statistical Analysis

Cecal communities were compared using Metastats (White et al., 2009) which is based on a non-parametric *t*-test, used to identify OTUs associated with carriage status (*p* < 0.05). Significant OTUs, with abundance higher than 0.001% in the community (Stanley et al., 2014) were identified in all experimental samples. The correlation coefficient and r-squared calculated and trend lines were drawn using Excel (Langer and Microsoft Corporation, 2007).

## Results

### *Salmonella* Abundance in the Ceca of Experimentally Infected, Commercial Broiler Chickens

**FIGURE 1:**
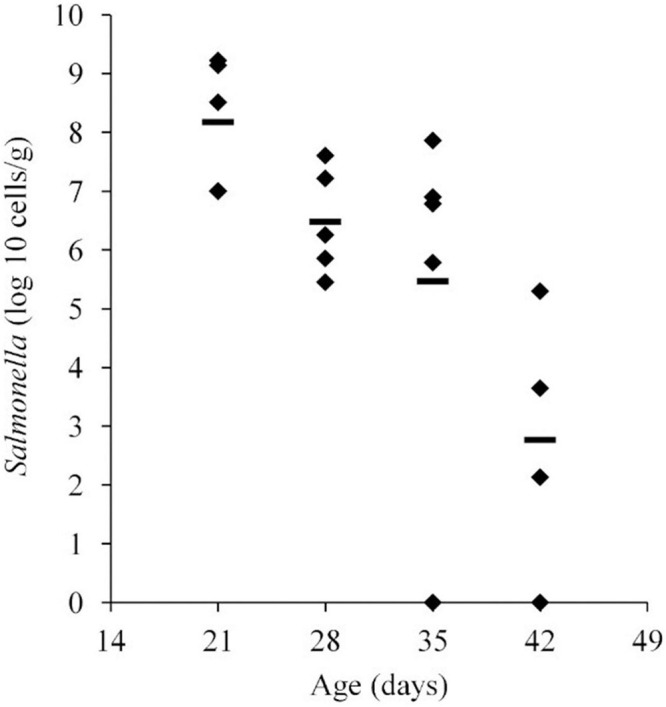
*Salmonella* abundance in cecal samples collected from chickens at 21, 28, 35, and 42 days old. Chickens were inoculated with 1.1 × 10^6^ CFU of *Salmonella* Typhimurium at 2 days of age. The mean abundance is indicated by the black line.

*Salmonella* abundance in the ceca was determined using qPCR for birds 21–42 days of age. The average *Salmonella* abundances were 6.8 × 10^8^ (range of 1.0 × 10^7^–1.6 × 10^9^), 1.2 × 10^7^ (range of 2.8 × 10^5^–4.0 × 10^7^), 1.7 × 10^7^ (range of 0–7.3 × 10^7^), and 5.1 × 10^4^ (range of 0–2.0 × 10^5^) CFU/g of ceca for broiler chickens at 21, 28, 35, and 42 days of age, respectively (Figure 1 and Table 1).

**TABLE 1:** *Salmonella* abundance in chicken ceca at 21, 28, 35, and 42 days old.

**Day**	**Isolator**^1^				
	**1**	**2**	**3**	**4**	**5**
21	8.51	7.00	9.14^2^	9.22^3^	7.00
28	5.85	7.20	5.45	6.00	7.60
35	6.89	6.79	0.00^4^	7.86	5.78
42	0.00^5^	2.11	3.64	5.30	–^6^

*^1^Log10 CFU/g.*

*^2^Permissive community 1.*

*^3^Permissive community 2.*

*^4^Exclusive community 1.*

*^5^Exclusive community 2.*

*^6^One bird left in isolator 5 died between days 35 and 42.*

### *Salmonella* Abundance Decreases in the Chicken Ceca With an Increase in Community Diversity

**FIGURE 2:**
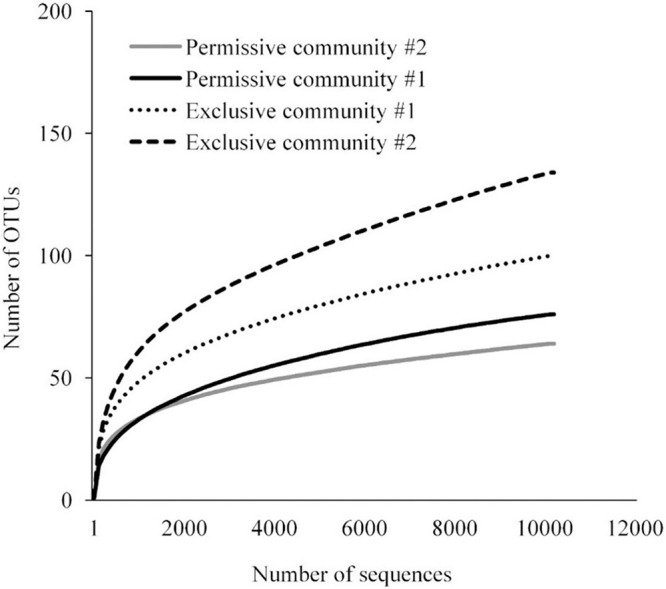
Rarefaction curve for bacterial 16S rRNA OTUs at 97% of similarity for *Salmonella* exclusive and permissive cecal communities.

A detailed data analysis was focused first on cecal communities with the two highest and two lowest *Salmonella* abundances; representing permissive and exclusive communities, respectively. Differences observed between exclusive and permissive communities were later applied across all samples. Cecal communities where no *Salmonella* was detected by PCR were designated as Exclusive communities 1 and 2; for days 35 (35-3) and 42 (42-1), respectively (See Table 1). Similarly, cecal samples with the two highest *Salmonella* abundance on day 21 (Permissive community 1: 1.4 × 10^9^; Permissive community 2: 1.7 × 10^9^ CFU/g) were designated as *Salmonella* permissive communities (See Table 1). Permissive community 1 and Exclusive community 2 resulted from birds removed from the same isolator. The region V3-V6 of the bacterial 16S rRNA was sequenced from the 19 cecal samples containing variable abundances of *Salmonella*. A total of 212,990 high quality filtered sequences were randomly selected; 11,210 16S rRNA sequences per cecal community. The sequences clustered into 485 OTUs. The average Good’s coverage, a method for estimating the percentage of the total species represented, was 99.7% ± 0.1% (mean ± SD); suggesting a sufficient sampling of the cecal bacterial communities. *Salmonella* exclusive cecal communities showed rarefaction curves with higher diversity compared to the permissive cecal communities (Figure 2). There were additional differences between exclusive and permissive communities as measured using several diversity parameters (Table 2). The number of OTUs, representing different bacterial species, was significantly (χ^2^ < 0.001) lower in permissive communities (70 ± 8) compared to exclusive communities (117 ± 24). Similarly, Chao index was significantly (χ^2^ < 0.001) lower in permissive communities (92.65 ± 0.21) in comparison to exclusive communities (172.05 ± 48.01). Species diversity was higher in the *Salmonella* exclusive cecal communities by the Shannon index (2.661 ± 0.20 versus 2.331 ± 0.06) and inverse Simpson index (7.277 ± 2.146 versus 7.103 ± 0.213). However, evenness was slightly higher in permissive communities (0.536 ± 0.006 versus 0.522 ± 0.006) by the Smith Wilson index.

**TABLE 2:** Diversity indices for *Salmonella* exclusive or permissive cecal communities.

**Community**	**Sobs**	**Chao**	**Shannon**	**Inverse Simpson**	**Smith and Wilson Evenness**
Permissive #1	76	92.8	2.291	6.953	0.532
Permissive #2	64	92.5	2.371	7.254	0.540
Exclusive #1	100	138.1	2.519	5.760	0.527
Exclusive #2	134	206.0	2.804	8.795	0.518

*Exclusive communities were PCR negative for *Salmonella* and permissive communities presented ceca with *Salmonella* abundance of 1 × 10^9^ CFU/g, as determined by qPCR.*

**FIGURE 3:**
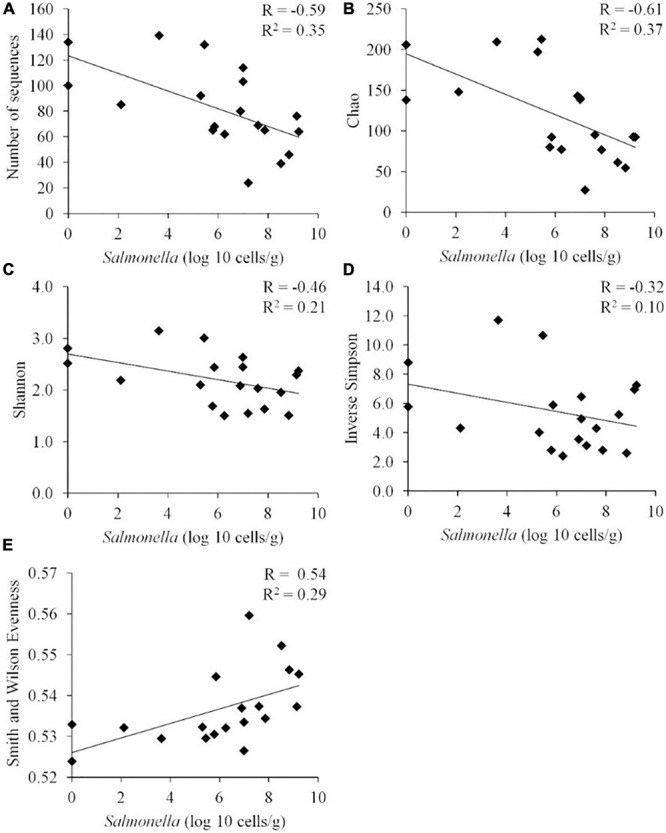
Correlation between the *Salmonella* abundance and community diversity for all cecal communities (*n* = 19). Community diversity was measured by number of unique OTUs **(A)** (*P* < 0.01), Chao index **(B)** (*P* < 0.01), Shannon index **(C)** (*P* < 0.01), inverted Simpson index **(D)** (*p* = 0.18), and Smith Wilson evenness index **(E)** (*P* < 0.02). *R*, Pearson correlation coefficient; and *R*^2^, coefficient of determination.

When these diversity indices were applied across all samples, a statistically significant negative correlation was observed between *Salmonella* abundance in the chicken ceca (expressed in logs) and the number of OTUs (*P* < 0.01), Chao index (*P* < 0.01), and Shannon index (*P* < 0.01) (*n* = 19) (Figure 3). While there appeared to be a negative correlation between *Salmonella* abundance and diversity, as measured by the inverse Simpson index, this correlation was not significant by the Pearson correlation coefficient (*p* = 0.18). Evenness presented a statistically significant positive correlation with the *Salmonella* cecal abundance (*P* < 0.02).

### Species Composition of *Salmonella* Exclusive and Permissive Cecal Communities and the Identification of Bacterial Species That May Influence *Salmonella* Abundance

Averaging across samples, the most abundant phyla observed in the cecal microbiota were *Firmicutes* (96.9%) and *Proteobacteria* (0.6%); and members of *Clostridiales* accounted for 90% of the total community 16S rRNA sequences (*n* = 193,003). The most abundant bacterial groups present in the ceca were unclassified *Lachnospiraceae* (37.2%), *Roseburia* (20.5%), *Clostridium* XI (13.5%), *Clostridium* XIVa (5.0%), *Blautia* (4.0%), unclassified *Firmicutes* (3.1%), unclassified *Ruminococcaceae* (3.0%), unclassified *Bacteria* (2.4%), unclassified *Clostridiales* (2.1%), *Faecalibacterium* (1.4%), *Oscillibacter* (0.5%), *Enterobacteriaceae* (0.5%), unclassified *Erysipelotrichaceae* (0.5%), unclassified *Bacillales* (0.4%), *Flavonifractor* (0.2%), and *Enterococcus* (0.2%).

**FIGURE 4:**
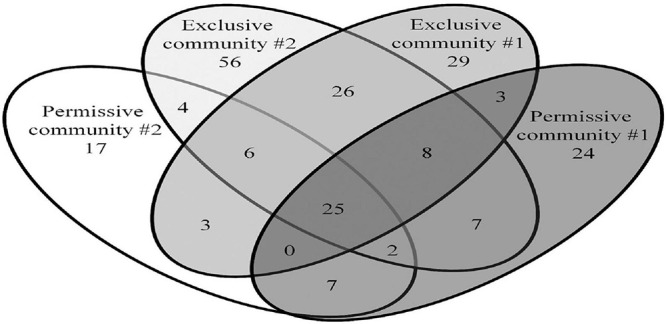
Shared and unique OTUs observed in *Salmonella* exclusive and permissive cecal communities. Numbers below groups indicate the number of OTUs.

The *Salmonella* exclusive community had a larger proportion of *Faecalibacterium* (*P* < 0.05), *Turicibacter* (*P* < 0.02), and unclassified *Firmicutes* (*P* < 0.005); and less *Blautia* (*P* < 0.01), and unclassified *Lachnospiraceae* (*P* < 0.05) compared to the permissive community. Twenty-six OTUs unique to exclusive communities were identified (Figure 4). These OTUs represent 6.99% of the total sequences for these cecal communities (*n* = 1,569). A smaller number of OTUs were unique to the permissive communities; 7 OTUs that represented 0.25% of the total 16S rRNA sequences (*n* = 58) (Table 2). A more diverse set of OTUs were observed in exclusive communities. The OTUs observed in permissive communities were affiliated with the phyla *Firmicutes* and *Proteobacteria*, while OTUs observed in exclusive communities belonged to the *Firmicutes*, *Proteobacteria* and *Actinobacteria* phyla. OTUs observed in permissive communities were related to the *Enterobacteriaceae*, *Enterococcaceae*, *Lachnospiraceae*, and *Clostridiaceae* families, while OTUs presented in exclusive communities were related to *Hyphomicrobiaceae*, *Eggerthellaceae*, *Clostridiaceae*, *Eubacteriaceae*, *Oscillospiraceae*, *Ruminococcaceae*, and *Lachnospiraceae* families (Table 3).

**TABLE 3:** OTUs unique to *Salmonella* exclusive or permissive cecal communities.

**Cecal community**		**Similarity to known bacterial species**			
	**Order**	**Family^1^**	**Genus and species**	**% Identity**	**OTU**
Permissive	*Enterobacteriales*	*Enterobacteriaceae*	*Salmonella enterica*	100	100
	*Lactobacillales*	*Enterococcaceae*	*Enterococcus faecalis*	100	113
	*Clostridiales*^2^	*Lachnospiraceae* (XIV)	*Blautia torques*	97	91
			*Murimonas intestini*	97	111
			*Robinsoniella peoriensis*	97	136
			*Blautia hansenii*	95	138
		*Ruminococcaceae* (III/IV)	*Ruminiclostridium leptum*	96	75
**% total 16s rRNA sequences (*n* = 58) = 0.25**					
Exclusive	*Eggerthellales*^3^	*Eggerthellaceae*	*Paraeggerthella hongkongensis*	97	115
	*Clostridiales*^2^	*Clostridiaceae* (I/II)	*Butyricicoccus pullicaecorum*	99	30
			*Flavonifractor plautii*	96	39
			*Clostridium grantii*	89	99
		*Eubacteriaceae* (XV)	*Eubacterium coprostanoligenes*	89	25
		*Lachnospiraceae* (XIV)	*Blautia luti*	97	198
			*Fusicatenibacter saccharivorans*	97	54
			*Coprococcus eutactus*	97	107
			*Syntrophococcus sucromutans*	98	118
			Tyzzerella propionicum	97	71
		*Oscillospiraceae*	*Oscillibacter ruminantium*	88	9
		*Ruminococcaceae* (III/IV)	*Gemmiger formicilis*	94	27
			*Ruminiclostridium leptum*	93	55
			*Ruminiclostridium leptum*	98	74
			*Ruminiclostridium leptum*	96	125
			*Ruminiclostridium leptum*	95	170
			*Acetanaerobacterium elongatum*	96	82
			*Ethanoligenens harbinense*	95	31
			*Ethanoligenens harbinense*	89	191
			*Ethanoligenens harbinense*	94	177
			*Papillibacter cinnamivorans*	95	36
			*Anaerotruncus colihominis*	100	58
			*Anaerobacterium chartisolvens*	90	155
			*Ruminiclostridium thermosuccinogenes*	89	89
			*Ruminococcus bromii*	94	289
			*Ruminococcus faecis*	96	190
**% total 16s rRNA sequences (*n* = 1,569) = 6.99**					

*^1^*Clostridia* phylogenetic cluster (Collins et al., 1994).*

*^2^*Firmicutes* phylum, *Clostridia* class.*

*^3^*Actinobacteria* phylum; *Coriobacteriia* class.*

**FIGURE 5:**
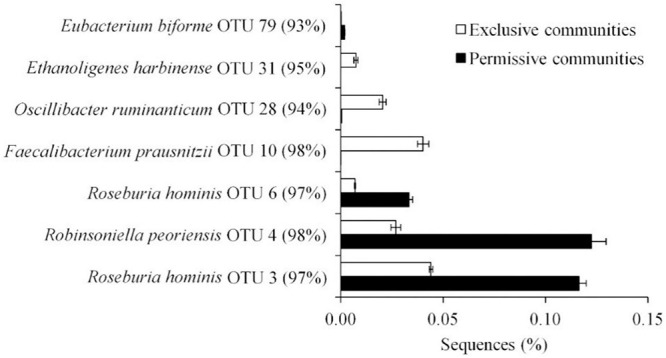
OTUs representing more than 0.01% of the total sequences and significantly (*P* < 0.05) associated with *Salmonella* exclusive and permissive cecal communities identified by Metastats.

**FIGURE 6:**
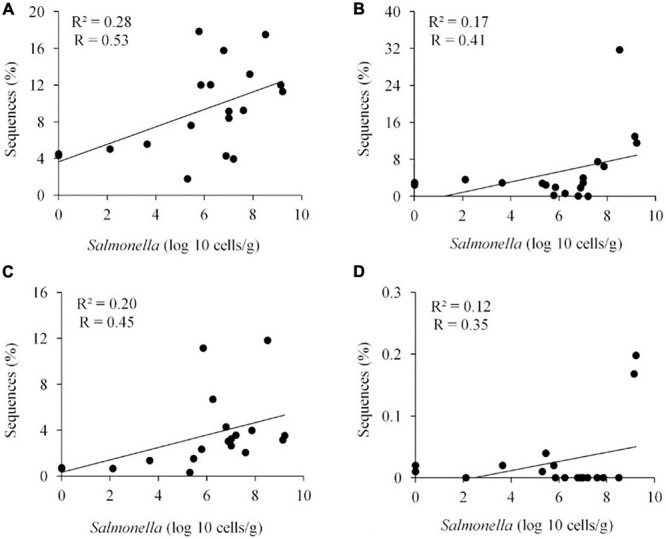
Positive correlation between the presence of specific OTUs and *Salmonella* abundance in the ceca (*n* = 19). *Roseburia hominis* OTU 3, 97% of similarity to RDP, *P* < 0.01 **(A)**; *Robinsoniella peoriensis* OTU 4, 98%, NS **(B)**; *Roseburia hominis* OTU 6, 97%, *P* < 0.05 **(C)**; and *Eubacterium biforme* OTU 79, 93%, NS **(D)**. *R*, Pearson correlation coefficient; and *R*^2^, coefficient of determination.

**FIGURE 7:**
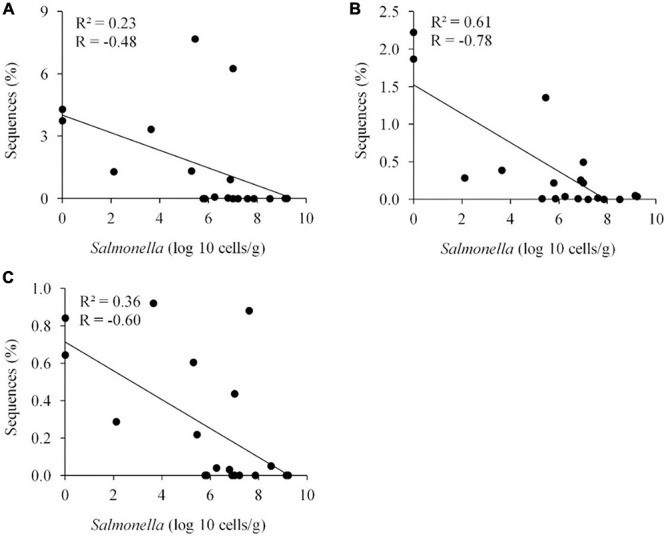
Negative correlation between the presence of specific OTUs and *Salmonella* abundance in the ceca (*n* = 19). *Faecalibacterium prausnitzii* OTU 10, 98%, *P* < 0.05 **(A)**; *Oscillibacter ruminantium* OTU 28, 95%, *P* < 0.001 **(B)**; and *Ethanoligenens harbinense* OTU 31, 95%, *P* < 0.01 **(C)**. *R*, Pearson correlation coefficient; and *R*^2^, coefficient of determination.

Metastats (Schloss et al., 2009) was used to quantify differences between groups and identify OTUs that most strongly influence the differences observed between the *Salmonella* permissive and exclusive communities (*p* < 0.05). Only OTUs with total abundance higher than 0.001% were considered in this analysis. Seven OTUs were identified (Figure 5). There was a greater frequency of OTUs related to *Eubacterium biforme* (OTU 79, 93% of similarity to RDP database), *Roseburia hominis* (OTU 6, presenting 97% of similarity), *Robinsoniella peoriensis* (OTU 4, 98% of similarity), and *Roseburia hominis* (OTU 3, 97% of similarity) in the *Salmonella* permissive communities. Of these 4 OTUs, there was a positive correlation between *Roseburia hominis* OTU 3 (97%, *P* < 0.01), and OTU 6 (97%, *P* < 0.05) with *Salmonella* abundance across all samples (*n* = 19) (Figure 6). These 4 OTUs accounted for 24.7% of the total 16S rRNA sequences for cecal samples with 10^9^ *Salmonella* cells/g and just 7.0% of the total sequences for *Salmonella*-negative cecal samples. *Ethanoligenens harbinense* (OTU 31, 95%), *Oscillibacter ruminantium* (OTU 28, 94%) and *Faecalibacterium prausnitzii* (OTU 10, 98%) were more abundant in the *Salmonella* exclusive cecal communities. There was a negative correlation between the presence of *Faecalibacterium prausnitzii* (OTU 10, 97% of similarity to RDP, *P* < 0.05), *Oscillibacter ruminantium* (OTU 28, 95%, *P* < 0.001) and *Ethanoligenens harbinense* (OTU 31, 95%, *P* < 0.01) with *Salmonella* abundance across all samples (*n* = 19) (Figure 7); and accounted for 6.1% of total 16S rRNA sequences for *Salmonella*, PCR-negative cecal samples. These OTUs represented just 0.04% of total sequences in cecal samples with 10^9^ *Salmonella* cells/g.

## Discussion

Unlike neonatal mammals, chicks seldom present symptoms of illness unless they are administered a large *Salmonella* challenge dose, at day of hatch. However, chicks orally administered *Salmonella* at 2 days of age exhibit little if any disease symptoms, and *Salmonella* abundance rapidly increases during the 1st week to 10^6^–10^7^ CFU/g and remains at these levels for 2–3 weeks. However, by the 4th week of age, there is a substantial decline in *Salmonella* abundance (Cheng et al., 2015). The composition of the intestinal community is also in flux up to the 3rd week of age when a distinct, stable community structure develops in the chicken ileum and ceca at approximately the same time that *Salmonella* abundance decreases (Lu et al., 2003a). Consequently, intestinal microbiota from chickens 21 days of age or older prevents *Salmonella* colonization in 8-day old layer chickens (Varmuzova et al., 2016). In this study, a wide difference in *Salmonella* abundance was observed in chickens at 35 and 42 days of age where 20% of birds were negative. This allowed the opportunity to compare intestinal community composition in birds with a range of *Salmonella* abundance.

Intestinal community diversity appears to adversely affect *Salmonella* abundance in chickens. Animals with highly diverse intestinal communities have been shown to be resistant to pathogen colonization and disease (Kamada et al., 2013). Bacterial diversity especially appears to be a significant factor affecting enteropathogen prevalence and abundance for shiga-toxin producing *Escherichia coli* (STEC) in cattle (Xu et al., 2014; Chopyk et al., 2016) and soil (van Elsas et al., 2012), *C. difficile* in humans (Antharam et al., 2013; Zhang et al., 2015), *C. perfringens* in chickens (Stanley et al., 2014), and *Campylobacter jejuni* in mice (Lone et al., 2013). In addition, community diversity seems to be an important predictor of intestinal health (Antharam et al., 2013; Wills et al., 2014; McMurtry et al., 2015; Rodriguez et al., 2015; Singh et al., 2015). A negative correlation between *Salmonella* abundance and evenness was also observed in the distribution of bacterial species. Evenness represents the degree to which species are distributed within a population. A lower evenness score for the *Salmonella* permissive community infers that a few bacterial species dominate the community. Similar findings have been reported for *Salmonella* in pigs and diarrheal illnesses in horses where lower evenness scores were associated with pathogen prevalence, abundance and symptoms of disease (Bearson et al., 2013; Rodriguez et al., 2015).

Intestinal community diversity appears to be a function of age, increasing as birds mature (Lu et al., 2003a; Crhanova et al., 2011; Videnska et al., 2013; Azcarate-Peril et al., 2018). The greatest diversity appears to be in the cecum, an intestinal compartment where *Salmonella* persists in the chicken (Azcarate-Peril et al., 2018). It was important to have all the birds exposed to the same treatment in order to control the possibility that *Salmonella* infection itself may alter the cecal community composition and species abundance (Videnska et al., 2013; Azcarate-Peril et al., 2018; Mon et al., 2020). With the exception of He et al. (2003), most studies did not observe a negative correlation between cecal community diversity and *Salmonella* abundance or prevalence (Crhanova et al., 2011; Videnska et al., 2013; Azcarate-Peril et al., 2018; Mon et al., 2020). In fact, one study observed an increase in species richness with *Salmonella* infection (Mon et al., 2020). It has been shown that changes in the intestinal community composition may result in a proteobacterial bloom favoring conditions of *Salmonella* proliferation (Singh et al., 2015; Zhang et al., 2015). *Salmonella* can also induce inflammation which reduces its obligate anaerobic competitors while providing it with additional nutrients for growth (Winter et al., 2010; Thiennimitr et al., 2011; Rivera-Chavez et al., 2016). While *Salmonella* may change the gut microbiome in chickens, it appears to involve a mechanism distinct from mammals (Rimet et al., 2019). However, these studies focused on chickens less than 21 days of age, early in chicken intestinal community development (Lu et al., 2003a). Varmuzova et al. (2016) demonstrated that only the intestinal microbiota from birds 21 days of age or older could reduce *Salmonella* colonization.

Specific genera and species were identified that correlated with *Salmonella* abundance in the chicken cecum. The majority of these OTUs were related to *Clostridiales*, the most abundant group of the chicken ceca (Lu et al., 2003a), with six OTUs identified as *Ethanoligenens harbinense*, *Oscillibacter ruminantium*, *Faecalibacterium prausnitzii*, *Roseburia hominis*, *Eubacterium biforme*, and *Robinsoniella peoriensis*. These genera or species have also been associated with enteropathogen colonization or intestinal health in other studies (Antharam et al., 2013; Miquel et al., 2013; Rossi et al., 2014; Stanley et al., 2014; Xu et al., 2014; Thibodeau et al., 2015; Knoll et al., 2016). Others have also noted absence or reduced abundance of *F. prausnitzii* and member species of the clostridial families (Collins et al., 1994) *Lachnospiraceae* (XIV) and *Ruminococcaceae* (III/IV) in birds colonized with *Salmonella* (Pourabedin et al., 2017; Liu et al., 2018; Khan and Chousalkar, 2020; Ding et al., 2021). Several of these species, associated with exclusive communities but absent from the *Salmonella* permissive community, may play an anti-inflammatory role in maintaining intestinal homeostasis and health (Wu and Wu, 2012; Miquel et al., 2013). Inflammation results in the production of tetrathionate and other metabolites which can be used to enhance *Salmonella* growth and thereby improve its persistence, and spread (Winter et al., 2010; Thiennimitr et al., 2011). However, these clostridial species also are likely to produce short chain fatty-acids (SCFA) such as butyrate, which reduces inflammation in the chicken intestine (Wu et al., 2016). Butyrate also represses expression of the *Salmonella* cell-invasion locus in SPI1 (Gantois et al., 2006) reducing its ability to elicit intestinal inflammation (Rivera-Chavez et al., 2016). *Clostridiales* abundance in the avian intestine may explain why birds are more resistant than mammals to *Salmonella* gastroenteritis.

Interestingly, several intestinal species may also have a positive impact on enteropathogen colonization (Xu et al., 2014; Thibodeau et al., 2015). Conceptually more emphasis is placed on pathogen exclusive species and mechanisms that explain their inhibitory effects, ignoring possible synergism between the pathogen and pathogen-permissive species as an alternate explanation behind competitive exclusion. The chicken intestinal microbiota can also produce fermentation end-products and other metabolites that can be metabolically exploited by *Salmonella* (Cheng et al., 2015). The metabolism of permissive species may result in cooperation with the pathogen, while the exclusive species may compete with the enteropathogen for these metabolites or out-compete the permissive species within the ecosystem. In either scenario, *Salmonella* would be unable to thrive in the chicken intestine.

While other studies have reported negative or positive correlations between intestinal community composition, abundance of specific genera/species and *Salmonella* abundance or prevalence (Videnska et al., 2013; Azcarate-Peril et al., 2018), this has been a tenuous association as reflected in comparisons among studies (Azcarate-Peril et al., 2018; Ma et al., 2020; Mon et al., 2020) and trials (Videnska et al., 2013). The exclusive genera/species, identified in this study, have been associated with *Salmonella* exclusion in some studies (Pourabedin et al., 2017; Liu et al., 2018; Khan and Chousalkar, 2020; Ding et al., 2021), but absent in others (Videnska et al., 2013; Azcarate-Peril et al., 2018; Ma et al., 2020; Mon et al., 2020). The genera/species, identified in this study, that negatively correlate with *Salmonella* are also absent in a competitive exclusion product known to effectively reduce *Salmonella* colonization in poultry (Pedroso et al., 2016). But the dominant member genera and species, present in this competitive exclusion product, are transient in chickens fed the product (Pedroso et al., 2016) indicating that the mechanism of competitive exclusion is complex. The contradictions may reflect the inherent nature of community diversity, which ensures there are always protagonists present to prevent enteropathogen colonization or illness in the animal population.

## Conclusion

Increased bacterial diversity and the composition of the cecal microbiota adversely affected *Salmonella* colonization in chickens. The isolation of the bacterial species associated with pathogen abundance are necessary to better understand the microbe-microbe interactions that exclude or permit pathogen persistence and a better understanding of the mechanism of competitive exclusion. Moreover, this work provides the fundamental first step toward the development of next generation, direct fed microbials that target and exclude enteropathogens from poultry.

## Data Availability Statement

All 16S rDNA sequence data is publicly available through National Center for Biotechnology Information (NCBI) the GenBank database under accession numbers KX913959 to KX914443.

## Ethics Statement

The animal study was reviewed and approved by the University of Georgia Animal Care and Use and Procedures Committee.

## Author Contributions

ML, AP, and JM: conceptualization and writing—review and editing. AP: methodology, formal analysis, and data curation. AP and JM: writing—original draft preparation. ML: supervision and project administration. ML and JM: funding acquisition. All authors have read and agreed to the published version of the manuscript.

## Conflict of Interest

The authors declare that the research was conducted in the absence of any commercial or financial relationships that could be construed as a potential conflict of interest.
